# Shaking culture improves physiological maintenance of primary rat kidney tissue slices

**DOI:** 10.3389/ftox.2026.1838970

**Published:** 2026-06-25

**Authors:** Moeno Kadoguchi, Kohei Matsushita, Jun Takahashi, Katsuhiro Esashika, Jingjing Yang, Masahiro Sugimoto, Ikumi Tamai, Hiroshi Arakawa

**Affiliations:** 1 Faculty of Pharmaceutical Sciences, Institute of Medical, Pharmaceutical and Health Sciences, Kanazawa University, Kanazawa, Ishikawa, Japan; 2 Division of Pathology, National Institute of Health Sciences, Kawasaki, Kanagawa, Japan; 3 Advanced Materials & Solutions Research Laboratory, Research Center, Mitsui Chemicals, Inc., Mobara-shi, Chiba, Japan; 4 Cell Culture Solution Department, New Business Incubation Center, Mitsui Chemicals, Inc., Chuo-ku, Tokyo, Japan; 5 Institute for Advanced Biosciences, Keio University, Tsuruoka, Yamagata, Japan; 6 Department of Regulatory Science, Graduate School of Pharmaceutical Sciences, Nagoya City University, Nagoya, Japan

**Keywords:** DIKI, kidney, renal transporter, shaking culture, tissue culture

## Abstract

**Introduction:**

Maintaining viability and physiological function in kidney tissue slice cultures remains a major challenge for *ex vivo* renal models, primarily due to limited oxygen diffusion into the tissue interior. In this study, we investigated whether shaking culture could improve the maintenance of primary rat kidney tissue slices.

**Methods:**

Rat kidney tissue slices (200 µm) were cultured under static or shaking conditions for up to 7 days. Tissue viability, histological features, renal transporter activity, nephrotoxic responses, and metabolomic profiles were evaluated.

**Results:**

Shaking culture markedly improved tissue viability, as evidenced by sustained ATP levels over 7 days compared with static conditions. Histological analysis revealed partial preservation of epithelial polarity, indicated by the maintained apical localization of aquaporin 1. Transporter studies demonstrated that uptake activities of Oat1/3 and Oct2 substrates were better preserved under shaking conditions. Furthermore, shaking-cultured slices enabled the evaluation of nephron segment–specific injury, with cisplatin inducing tubular damage and puromycin or dasatinib affecting glomerular structures. Metabolomic analysis revealed distinct alterations in central carbon metabolism, including increased levels of tricarboxylic acid cycle intermediates, suggesting improved mitochondrial function.

**Conclusion:**

These findings demonstrate that shaking culture provides a simple and effective approach to improve the culture microenvironment, thereby improving the maintenance of viability and physiological function in kidney tissue slices. This system may serve as a useful platform for investigating renal pharmacokinetics and drug-induced kidney injury.

## 1 Introduction

The kidney plays a central role in maintaining systemic homeostasis by regulating electrolyte balance, acid-base status, fluid volume, and the elimination of endogenous and xenobiotic compounds. It is a structurally complex organ composed of multiple specialized cell types, including glomerular cells responsible for filtration and tubular epithelial cells that carry out energy-intensive transport processes underlying the reabsorption and secretion of a wide range of solutes. In addition, other nephron segments, such as the loop of Henle, distal tubules, and collecting ducts, as well as interstitial cells, contribute to the coordinated regulation of renal function. Beyond its physiological importance, the kidney is a major organ involved in drug disposition and a frequent target of adverse drug reactions. This structural and functional complexity underlies the difficulty of reproducing renal physiology *in vitro*.

Experimental systems that enable mechanistic investigation of renal function are important in drug discovery and development, as renal transport processes are essential determinants of both pharmacokinetics and drug-induced toxicity. However, the development of physiologically relevant *in vitro* kidney models remains challenging (Arakawa et al., 2025). Conventional cell culture systems, including immortalized renal cell lines, often fail to capture the structural complexity and functional heterogeneity of the nephron. In particular, these models poorly reproduce the coordinated functions of multiple nephron segments and the transporter-mediated processes that are critical for renal drug handling.

Kidney tissue slices represent a promising experimental platform to address these limitations. Tissue slices preserve the native architecture of the kidney, including multiple nephron segments and diverse cell types within a shared tissue environment. This structural preservation enables investigation of renal transport processes and drug responses under conditions that more closely resemble those of the *in vivo* kidney. Despite these advantages, maintaining tissue viability and physiological function in kidney slice cultures remains technically challenging. A major limitation of organ slice culture systems is the limited diffusion of oxygen into the interior of the slice, reducing oxygen availability for cells located deeper within the tissue during culture. This diffusion limitation has been widely recognized in organ slice models of several tissues and represents a critical determinant of tissue survival and metabolic activity.

Adequate oxygen supply is particularly critical for kidney tissue, as renal tubular epithelial cells rely heavily on mitochondrial oxidative metabolism to generate the ATP required for active solute transport (Bhargava and Schnellmann, 2017). Insufficient oxygen delivery *in vitro* can therefore lead to rapid ATP depletion, loss of transporter activity, and deterioration of tissue viability. Several approaches have been proposed to improve oxygen availability in organ slice cultures, including oxygen-permeable culture substrates (Arakawa et al., 2022; Kadoguchi et al., 2026; Danoy et al., 2022; Nishikawa et al., 2022; Takemura et al., 2023; Ito et al., 2025) that enhance medium mixing. Among these strategies, shaking culture may represent a simple and practical method to improve oxygen supply by reducing diffusion limitation at the medium-tissue slice interface.

In the present study, we investigated whether shaking culture could improve the maintenance of viability and physiological function in primary rat kidney tissue slices. We evaluated tissue viability and functional activity in kidney tissue slices, suggesting that dynamic culture conditions provide a more favorable microenvironment for *ex vivo* renal tissue culture.

## 2 Materials and methods

### 2.1 Materials

Cisplatin (cis-Diammineplatinum(II) Dichloride), dasatinib, and probenecid were purchased from Tokyo Chemical Industry (Tokyo, Japan). Cimetidine and puromycin aminonucleoside were purchased from FUJIFILM Wako Pure Chemical Corporation (Osaka, Japan). [3H]p-aminohippuric acid (PAH) and [14C]metformin were purchased from PerkinElmer (Waltham, MA, United States). Rabbit monoclonal anti-WT1 antibody (ab267377) and goat polyclonal anti-rabbit IgG (ab150081) were purchased from Abcam (Cambridge, United Kingdom). Mouse monoclonal anti-aquaporin 1 (Aqp1) antibody (clone: B-11; sc-25287) was purchased from Santa Cruz Biotechnology (Dallas, TX, United States). Goat polyclonal anti-kidney injury molecule-1 (Kim-1) antibody (AF3689) was purchased from R&D Systems (Minneapolis, MN, United States). Histofine Simple Stain MAX PO was purchased from Nichirei Bioscience, Inc. (Tokyo, Japan). The InnoCell™ non-treated 24-well plates were provided by Mitsui Chemicals, Inc. (Tokyo, Japan).

### 2.2 Animal

Male Wistar rats (7–9 weeks old) were purchased from Sankyo Labo Service Corporation Inc. (Hamamatsu, Japan). The rats were housed under controlled conditions (3 rats per cage, 12 h light/dark cycles, temperature 24.0 °C ± 1 °C, humidity 55% ± 5%) and had free access to water and a standard diet throughout the experimental period. The Kanazawa University Institutional Animal Care and Use Committee and the Animal Experiment Ethics Committee of Nagoya City University Graduate School of Pharmaceutical Sciences approved all animal experiments. All protocols for animal experiments performed in the present study were approved by the committee (Approval Number: AP23-049 for Kanazawa University and 25-008H03 for Nagoya City University).

### 2.3 Primary culture of rat kidney tissue slices

Rat kidney tissue slices (200 µm thick) were prepared using a microslicer (Zero 1; Dosaka EM, Kyoto, Japan) as described previously (Kadoguchi et al., 2026). The slices were transferred into InnoCell™ non-treated 24-well plates prewarmed to 37 °C, containing 300 µL of Williams’ Medium E (Thermo Fisher Scientific, Waltham, MA, United States) supplemented with 5% (v/v) fetal bovine serum (Serana Europe GmbH, Brandenburg, Germany), 100 units/mL penicillin-streptomycin (Nacalai Tesque, Kyoto, Japan), 0.1% (v/v) dexamethasone, 1% (v/v) ITS Liquid Media Supplement (Sigma-Aldrich, St. Louis, MO, United States), and 2 mM alanylglutamine (FUJIFILM Wako Pure Chemical Corporation) in the presence or absence of the test drugs. The slices were incubated at 37 °C in a humidified atmosphere containing 5% CO_2_ for up to 7 days, with the medium changed daily. The drugs were replenished with each medium replacement. Shaking culture was performed by incubating the culture plates on an orbital shaker (MINI WAVE, AS ONE Corporation, Osaka, Japan) placed inside a CO_2_ incubator at the indicated rotational speeds (0, 10, 50, 100, 150 rpm), with 0 rpm used as the static control.

### 2.4 ATP assay

ATP content of rat kidney tissue slices was measured using the “Tissue” ATP Assay Kit (TOYO B-Net Co., Ltd., Tokyo, Japan) as described previously (Arakawa et al., 2022; Kadoguchi et al., 2026). Luminescence was measured using a microplate reader (Infinite200PRO; Tecan Group Ltd., Männedorf, Switzerland). ATP content of the slices was normalized to slice weight.

### 2.5 Uptake study

Rat kidney tissue slices were pre-incubated with fresh transport buffer for 5 min at 37 °C or 4 °C under oxygen bubbling. The transport buffer was then replaced with 1 mL of transport buffer containing the test drug, and the mixture was incubated for 15 min under oxygen bubbling. Uptake was terminated by washing the slices three times with ice-cold transport buffer. Slice weights were measured, and the slices were dissolved in 1N NaOH at 60 °C. After neutralization with 5N HCl, radioactivity was measured using a liquid scintillation counter (LSC-7200, ALOKA Co., Ltd., Tokyo, Japan).

### 2.6 Histopathological analysis

Histopathological procedures were described previously (Kadoguchi et al., 2026), with minor modifications. Rat kidney tissue slices were fixed with 10% Formaldehyde Neutral Buffer Solution (Nacalai Tesque) or methanol. The kidney tissue slices were embedded in paraffin and sectioned at 4 µm for hematoxylin-eosin (H&E) staining. For immunohistochemistry, sections were autoclaved for antigen retrieval, followed by blocking with 10% normal goat serum (AQP1) or rabbit serum (Kim-1), and then incubated at 4 °C overnight with antibodies targeting AQP1 (1:300) and Kim-1 (1:1,000). Subsequently, the sections were incubated with a horseradish peroxidase-conjugated antibody. The antibody reaction was visualized using 3,3′-diaminobenzidine tetrahydrochloride. For WT-1 immunofluorescence staining, slices were incubated with 1% bovine serum albumin, followed by incubation with a rabbit monoclonal anti-WT1 antibody (1:100) overnight 4 °C. Subsequently, the slices were incubated with goat polyclonal anti-rabbit IgG antibody for 2 h at room temperature. Fluorescence images were acquired using BZ-X810 (Keyence, Osaka, Japan).

### 2.7 Metabolome analysis

Rat kidney tissue slices cultured under static and shaking conditions for 3 days were weighed into screw tubes and homogenized with zirconia beads (5 mm × 2, 3 mm × 4) in methanol containing the internal standards, D-camphor-10-sulfonic acid (CSA), 2-(N-morpholino)ethanesulfonic acid, and methionine sulfate. The detailed procedure was described previously (Kajiwara et al., 2024; Ohta et al., 2024). Briefly, absolute quantification was performed using capillary electrophoresis–time-of-flight-mass spectrometry (CE-TOFMS). All samples were analyzed in a single batch to minimize potential batch effects. A mixture of standard compounds was measured within the same batch. Internal standards were included in both the biological samples and the standard mixture, and signal intensities were normalized using these internal standards to correct for run-to-run variation in MS sensitivity. Migration times in the CE system were also corrected using internal standards. Metabolite identification was performed based on matching authentic standards. *m/z* values were matched within a 10 ppm tolerance, and migration times were aligned and matched within 0.1 s after correction using internal standards. Isotopic patterns were also examined to exclude noise-derived signals. Metabolites that met these criteria were identified at MSI Level 1. It was confirmed that most detected peaks were within the linear range of the calibration curves.

### 2.8 Statistical analysis

Data are presented as the mean ± standard deviation (S.D.). Statistical significance was evaluated using Student’s t-test or ANOVA, followed by a Tukey-Kramer test. A *p* < 0.05 was considered statistically significant. Principal component analysis (PCA) and pathway analysis were performed using MetaboAnalyst ver. 6.0 (https://www.metaboanalyst.ca/). For PCA, metabolite concentration data were log10-transformed and auto-scaled without sample normalization prior to analysis. For pathway analysis, quantitative metabolite concentration data were uploaded as a concentration table without additional normalization, transformation, or scaling. Pathway enrichment analysis was conducted using the Kyoto Encyclopedia of Genes and Genomes (KEGG) pathway library for *Rattus norvegicus* (rat) using the quantitative enrichment analysis (QEA) framework together with pathway topology analysis. MetaboAnalyst-generated pathway visualization plots were based on raw p-values according to the default software setting, while false discovery rate (FDR)-adjusted p-values were also calculated and considered during data interpretation.

## 3 Results

### 3.1 Shaking culture preserves viability of rat kidney tissue slices

**FIGURE 1:**
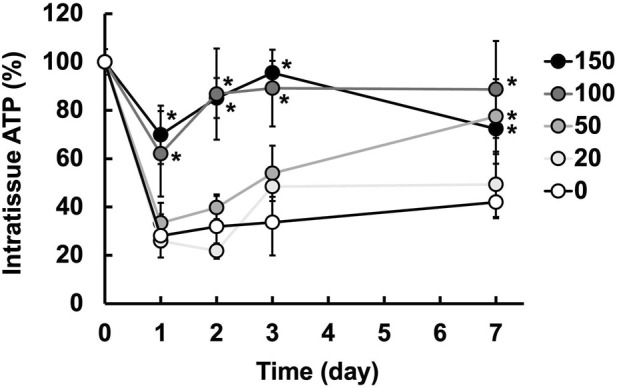
Viability of rat kidney tissue slices cultured under static and shaking conditions. Rat kidney tissue slices were cultured under static or shaking conditions (0, 20, 50, 100, 150 rpm) for 7 days. Tissue viability was assessed by measuring ATP content. Data are represented as mean ± S.D. (n = 3–6). * indicates statistically significant differences from the static conditions (*p* < 0.05, Tukey-Kramer test).

The effects of shaking culture on the viability of primary cultured rat kidney tissue slices were evaluated. Tissue slices were cultured for 7 days under static (0 rpm) or shaking conditions (20, 50, 100, and 150 rpm), and viability was evaluated by measuring intratissue ATP levels. ATP levels in slices cultured at 100 or 150 rpm remained comparable to those measured immediately after isolation throughout the 7-day culture period (Figure 1). Based on these results, subsequent experiments were performed at 100 rpm.

### 3.2 Effects of shaking culture on the histological features of kidney tissue slices

**FIGURE 2:**
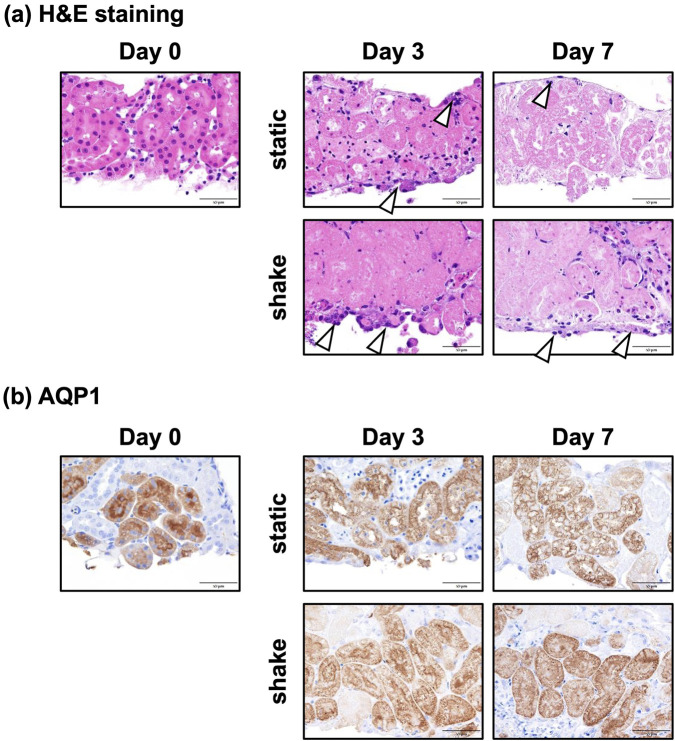
Histological features of primary cultured rat kidney tissue in static and shaking conditions. Histological analysis **(a)** and immunohistochemical staining for aquaporin 1 (AQP1) **(b)** were performed on primary cultured rat kidney tissue slices under static and shaking conditions for 3 and 7 days. Arrowheads indicate regenerative tubules.

The effects of shaking culture on tissue structure and protein localization were examined by histological analysis. Kidney tissue slices were cultured under static and shaking conditions for 3 or 7 days, then stained with H&E and immunostained for AQP1. H&E staining revealed no remarkable differences in tissue structure between the static and shaking culture conditions (Figure 2a). In both culture conditions, regenerated tubules were observed on the cut surface of the tissue slice. Immunohistochemistry of AQP1 showed that AQP1 expression gradually decreased with increasing culture duration under both culture conditions over 7-day culture period (Figure 2b). However, slices cultured under shaking conditions maintained relatively stronger apical localization of AQP1 compared with slices cultured under static conditions (Figure 2b). These results suggest that shaking culture better preserves proximal tubular polarity during culture.

### 3.3 Effects of shaking culture on drug transporter activity in primary cultured rat kidney tissue slices

To examine the effects of shaking culture on renal drug transporter activities, uptake studies were performed using Oat1/3 substrate PAH and Oct2 substrate metformin. In freshly isolated slices (Day 0), PAH uptake was 3.56 ± 0.85 μL/mg kidney. Co-incubation with probenecid, an Oat1/3 inhibitor, significantly reduced PAH uptake to 1.56 ± 0.13 μL/mg kidney, confirming functional Oat1/3 transport activity. After 2 days of static culture, PAH uptake decreased to 1.05 ± 0.18 μL/mg kidney, and uptake levels were similar to those observed in the presence of probenecid and 4 °C, indicating loss of transport activity. In contrast, slices cultured under shaking conditions for 2 days exhibited higher PAH uptake (1.62 ± 0.19 μL/mg kidney), which was significantly reduced to 1.10 ± 0.13 μL/mg kidney by probenecid (31.8%), suggesting that shaking culture partially preserves Oat1/3-mediated transport activity.

**FIGURE 3:**
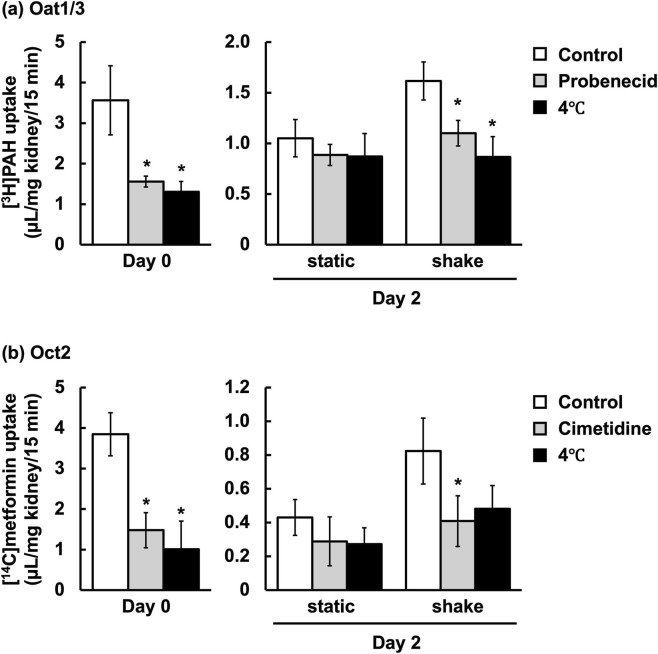
Transporter-mediated uptake in primary cultured rat kidney tissue slices. Uptake of [^3^H]PAH (45 nM; 0.18 μCi/mL) **(a)** and [^14^C]metformin (60 nM; 0.18 μCi/mL) **(b)** by primary cultured rat kidney tissue slices in static and shaking (100 rpm) conditions. Uptake was evaluated in the absence or presence of probenecid (1 mM) or cimetidine (1 mM) at 37 °C or 4 °C for 15 min. Data are represented as mean ± S.D. (n = 3–6). * indicates statistically significant differences from the control group (*p* < 0.05, Tukey-Kramer test).

Metformin uptake showed a similar trend. In freshly isolated slices, metformin uptake was 3.85 ± 0.53 μL/mg kidney and was significantly reduced to 1.48 ± 0.43 μL/mg kidney (Figure 3a) by co-incubation with the Oct2 inhibitor cimetidine, confirming functional Oct2 transport activity. After 2 days of static culture, metformin uptake was not significantly different from that observed in the presence of cimetidine or 4 °C. In contrast, slices cultured under shaking conditions exhibited metformin uptake (0.82 ± 0.20 μL/mg kidney), which was significantly reduced to 0.41 ± 0.15 μL/mg kidney by cimetidine (Figure 3b). Although transporter activity decreased compared with freshly isolated slices, these results suggest that shaking culture better preserves drug transporter activity than static culture.

### 3.4 DIKI evaluation using rat kidney tissue slices cultured under shaking conditions

**FIGURE 4:**
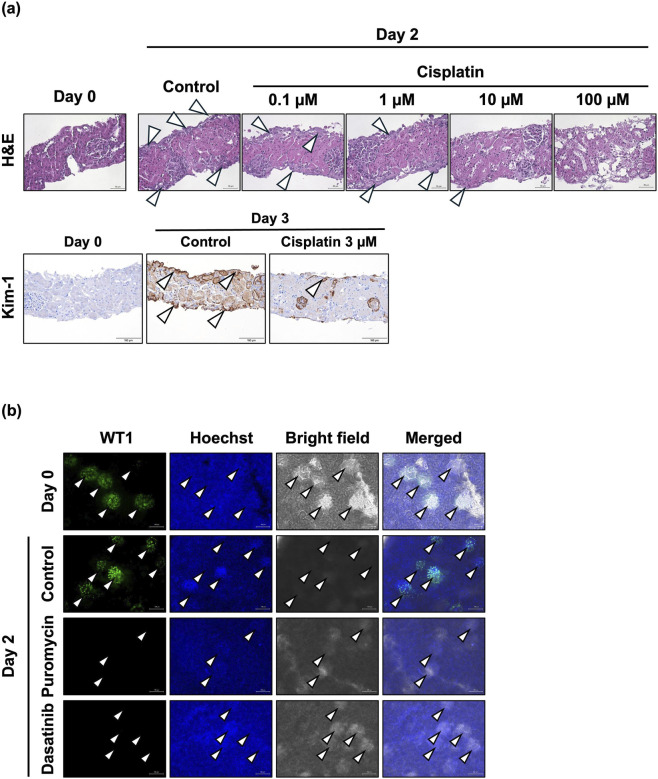
Evaluation of cisplatin-, puromycin-, and dasatinib-induced kidney injury by primary rat kidney tissue slices. **(a)** H&E staining and immunohistochemical staining for Kim-1 in primary cultured rat kidney tissue slices in absence or presence of cisplatin (0.1, 1, 3, 10, 100 μM) for 2 or 3 days. White arrowheads indicate regenerative tubules. **(b)** Immunofluorescence staining for WT1 in primary cultured rat kidney tissue slices in the absence or presence of puromycin (100 µM) and dasatinib (2 µM) for 2 days. Nuclei were counterstained with Hoechst33342. White arrowheads indicate glomeruli.

To evaluate DIKI across different nephron segments, kidney slices were exposed for 2 days to cisplatin, which induces tubular toxicity, and to puromycin and dasatinib, which induce glomerular toxicity. Subsequently, H&E staining and immunostaining were performed. In the cisplatin group, H&E staining revealed concentration-dependent tissue disruption (Figure 4a). In addition, the number of regenerative tubules observed at the cut surface of the tissue slices decreased in a cisplatin concentration-dependent manner. In particular, the number of regenerative tubules decreased with increasing cisplatin concentrations from 1 to 10 µM. Therefore, Kim-1 immunostaining, a tubule regeneration marker, was performed at the intermediate concentration of 3 µM because this condition was considered suitable for clearer histological evaluation of tubular regenerative changes. Both H&E staining and Kim-1 immunostaining revealed decreased regenerated tubular structures following cisplatin exposure. These findings suggest that cisplatin suppressed tubular regeneration as a consequence of tubular injury. For the evaluation of glomerular toxicity, immunostaining for Wilms’ tumor-1 (WT1), a nuclear transcription factor involved in maintaining the glomerular structure and function (Palmer et al., 2001; Guo, 2002; Wagner et al., 2004), was performed after exposure to puromycin and dasatinib (Figure 4b). Changes in WT1 staining indicated glomerular injury in these treatment groups. Collectively, these results demonstrate that rat kidney tissue slices cultured under shaking conditions allow simultaneous evaluation of nephron segment–specific injury.

### 3.5 Shaking culture alters the metabolic profiles of kidney tissue slices

**FIGURE 5:**
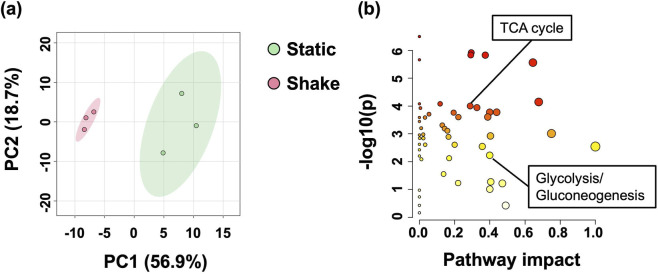
Metabolome analysis of primary cultured rat kidney tissue slices. **(a)** Principal component analysis (PCA) of metabolomic profiles in primary cultured rat kidney tissue slices. Each dot represents an individual kidney tissue slice sample. Colors indicate experimental groups, static (green) and shaking (red). Each dot represents an individual sample. PC1 (56.9%) and PC2 (18.7%) indicate the proportion of total variance explained by each principal component. The shaded areas represent the 95% confidence intervals for each group (static and shaking conditions). The kidney tissue slice samples shown in the PCA plots represent technical replicates derived from the same biological samples, in which individual kidney tissue slices obtained from the same animal were cultured independently in separate wells. **(b)** Pathway significantly altered in primary cultured kidney tissue slices between static and shaking conditions. The x-axis indicates pathway impact, and the y-axis shows statistical significance expressed as −log10 (p-value). Each dot represents a metabolic pathway.

To explore the metabolic changes associated with shaking culture, metabolic analysis was performed on rat kidney tissue slices under static and shaking conditions for 2 days. As a result, a total of 134 metabolites were detected (Supplementary Table S1). Samples from the shaking and static culture conditions were clearly separated in the score plot (Figure 5a). PCA revealed a clear separation between static and shaking conditions along PC1 (56.9%), indicating that culture conditions were the primary source of metabolic variation (Figure 5a). Each point represents an individual sample. Notably, samples under static conditions exhibited greater dispersion than those under shaking conditions, suggesting increased metabolic variability. In contrast, samples from the shaking conditions were more tightly clustered, indicating improved reproducibility and more stable metabolic profiles.

Compared with static culture, 42 metabolites were significantly increased, and 5 metabolites were significantly decreased under shaking culture conditions after FDR correction (Supplementary Figure S1). Pathway analysis indicated significant alterations in pathways related to central carbon metabolism (Figure 5b; Supplementary Table S2). In particular, the tricarboxylic acid (TCA) cycle intermediates malate and fumarate were significantly higher in slices cultured under shaking conditions than in static culture. Although the glycolysis/gluconeogenesis pathway did not reach statistical significance at the pathway level (P = 0.060), several metabolites associated with this pathway were significantly altered under shaking culture conditions. Specifically, the levels of 3-phosphoglycerate (3PG), glucose-6-phosphate (G6P), and glucose-1-phosphate (G1P) were significantly higher than in static culture. These findings suggest that shaking culture may influence central carbon metabolism.

## 4 Discussion

Maintaining viability and physiological function in kidney tissue slice cultures remains a major challenge for *ex vivo* renal models. In the present study, shaking culture markedly improved the preservation of rat kidney tissue slices, as demonstrated by sustained ATP levels, partial maintenance of renal transporter activity, preserved responsiveness to nephrotoxic drugs, and metabolic changes associated with energy metabolism. These results indicate that dynamic culture conditions provide a more favorable microenvironment for maintaining functional kidney tissue slices compared with static culture.

First, shaking culture significantly improved the maintenance of tissue viability, as evidenced by sustained ATP levels for up to 7 days (Figure 1). Our previous study using oxygen-permeable plates under static culture conditions partially attenuated ATP depletion in kidney tissue slices (Arakawa et al., 2022; Kadoguchi et al., 2026). However, even with oxygen-permeable plates, ATP levels remained at approximately 50% of those observed in freshly isolated kidney tissues, indicating that oxygen-permeable plates alone were insufficient to fully preserve tissue viability during prolonged culture. In contrast, the present study demonstrated that combining oxygen-permeable plates with shaking culture maintained intratissue ATP levels throughout the 7-day culture period. These findings suggest that shaking culture may complement oxygen-permeable culture systems by further improving the culture microenvironment. Renal tubular epithelial cells have high metabolic demands and rely heavily on mitochondrial oxidative phosphorylation to generate the ATP required for active solute transport (Bhargava and Schnellmann, 2017). In organ slice culture systems, oxygen must diffuse from the culture medium into the interior of the slice, which can limit oxygen availability in deeper regions of the tissue. The effective oxygen diffusion distance in biological tissues is generally estimated to be approximately 100–300 µm (De Graaf et al., 2010; Fisher and Vickers, 2013; Humpel, 2015; Neuhaus et al., 2017; Dewyse et al., 2021; Preuß et al., 2022; Siwczak et al., 2023). Therefore, cells located farther from the slice surface may experience reduced oxygen availability under static culture conditions. The improved ATP maintenance observed under shaking conditions suggests that dynamic culture may enhance oxygen and nutrient supply by promoting medium mixing and reducing diffusion limitations at the medium-tissue slice interface.

Slice thickness is another important factor affecting oxygen diffusion and tissue viability in organ slice cultures. In the present study, kidney tissue slices with a thickness of 200 µm were used. In preliminary experiments, thinner slices (approximately 100 µm) were also prepared; however, regions lacking glomeruli were frequently observed, indicating that thin slices may not adequately represent the full renal microarchitecture. Moreover, the thinner slices were more fragile and prone to mechanical damage during handling. These observations suggest that although thinner slices may theoretically improve oxygen and drug diffusion, excessively thin slices may compromise structural integrity and experimental reproducibility. Therefore, the use of 200 µm slices likely represents a practical balance between maintaining tissue architecture and ensuring adequate oxygen supply.

Histopathological analysis indicated that shaking culture partially preserved tissue structure and epithelial polarity (Figure 2a). Although overall tissue architecture was maintained under both culture conditions, apical localization of AQP1 was relatively better preserved in slices cultured under shaking conditions (Figure 2b). Renal tubular epithelial cells possess highly polarized structures that are essential for vectorial solute transport, and disruption of cellular polarity is commonly associated with ATP depletion and cytoskeletal disorganization in proximal tubular cells (Bush et al., 2000; Schlüter and Margolis, 2012). Therefore, the improved preservation of ATP levels in kidney tissue slices under shaking culture conditions may partly contribute to the maintenance of epithelial polarity during *ex vivo* culture.

In addition to structural preservation, shaking culture also partially maintained renal drug transporter activity (Figure 3). Because renal transporter function is closely associated with transporter-mediated nephrotoxicity, preservation of transporter activity during culture is important for evaluating nephrotoxic responses in kidney tissue slices. Our previous study demonstrated that nephrotoxic responses were more clearly detectable after 48-h exposure than after 24-h exposure (Arakawa et al., 2022). Therefore, maintenance of transporter activity for at least 2 days was considered important for transporter-related nephrotoxicity assessment in cultured kidney tissue slices. Transporters such as OAT1/3 and OCT2 are key determinants of renal drug secretion and play important roles in renal pharmacokinetics and drug-induced nephrotoxicity (Yokoo et al., 2007; Filipski et al., 2009; Ciarimboli et al., 2010; Nakamura et al., 2010; Sauzay et al., 2016; Uwai et al., 2007; Bam et al., 2014; Arakawa et al., 2025). Loss of transporter activity during culture is a well-known limitation of kidney slice models. In the present study, the uptake of PAH, an Oat1/3 substrate, and metformin, an Oct2 substrate, was better preserved in slices cultured under shaking conditions than in static culture, suggesting that dynamic culture conditions may help maintain transporter activity. The uptake activities after culture were approximately 46% of Day 0 levels for PAH and approximately 21% for metformin under shaking conditions, indicating a partial decline in transporter function during culture. In addition, AQP1 immunostaining showed reduced apical localization after culture compared with Day 0. These findings suggest that, although overall tissue viability was maintained, tubular epithelial cell differentiation and polarity were only partially preserved under the current culture conditions. Because transporter localization and activity are closely associated with epithelial polarity, disruption of tubular architecture during culture may have contributed to the reduced transporter function observed after prolonged incubation. Nevertheless, transporter-mediated uptake remained clearly detectable and significantly higher than that observed under static conditions. These findings suggest that the shaking culture method may be useful for comparative or qualitative evaluation of transporter-related responses in kidney tissue slices, although further optimization of culture conditions will be necessary to better preserve transporter localization and activity during prolonged culture.

Importantly, the present kidney tissue slice culture system also enables the evaluation of nephron segment-specific injury. For nephrotoxicity assessment, kidney tissue slices were exposed to test compounds for 48 h because decreases in intratissue ATP levels were not consistently observed after 24-h exposure, whereas exposure for 48 h or longer enabled detection of toxic responses. Exposure to cisplatin resulted in concentration-dependent inhibition of tubular regeneration (Figure 4a), whereas puromycin and dasatinib induced alterations in WT1 staining (Figure 4b), indicative of glomerular injury (Rico et al., 2005; Yan et al., 2012; Shen et al., 2016; Calizo et al., 2019). These findings suggest that kidney tissue slices cultured under shaking conditions retain sufficient structural and functional integrity to allow simultaneous assessment of tubular and glomerular toxicity. Because many forms of DIKI involve segment-specific mechanisms (Ricci and Ronco, 2012), the ability to evaluate different nephron segments within a single experimental system represents a key advantage of this model. However, further validation using a broader range of compounds and additional glomerular functional markers, such as nephrin and podocin, will be necessary. Moreover, ultrastructural analysis using electron microscopy may further clarify glomerular structural alterations in cultured kidney tissue slices. On the other hand, although albumin leakage is widely used as a clinical indicator of glomerular dysfunction, direct evaluation of albumin leakage is difficult in kidney tissue slice culture systems because glomerular filtration cannot be fully reproduced under the present *ex vivo* culture conditions. Furthermore, direct comparison of glomerular injury responses between static and shaking culture conditions was not performed in the present study. Therefore, although the present study demonstrated that glomerular injury responses could be evaluated under shaking culture conditions, the superiority of shaking culture for glomerular toxicity assessment remains to be established. Overall, these findings suggest the potential utility of shaking cultured kidney tissue slices as a platform for evaluating renal toxicity and pharmacokinetics.

Metabolomic analysis revealed alterations in central carbon metabolism under shaking culture conditions. Day 3 was selected for metabolomic analysis to better characterize metabolic differences between static and shaking culture conditions. At this time point, differences in intratissue ATP levels between the two culture conditions were more pronounced while the tissue still retained measurable viability during the extended culture period. Concentrations of TCA cycle intermediates, such as malate and fumarate, were increased (Figure 5b; Supplementary Tables S1, S2). These findings suggest that mitochondrial metabolic activity may be better maintained under shaking conditions. Because mitochondrial oxidative metabolism is essential for ATP production in renal tubular cells, improved maintenance of mitochondrial metabolism may contribute to the sustained viability observed in shaking culture. In the PCA score plots, samples cultured under static conditions exhibited greater dispersion than those under shaking conditions, indicating greater metabolic heterogeneity. This variability may reflect uneven oxygen and nutrient distribution within the tissue slices under static conditions, leading to heterogeneous metabolic states across samples. In contrast, the tighter clustering observed under shaking conditions suggests a more uniform metabolic environment, likely due to improved medium mixing and oxygen availability. Such a reduction in metabolic variability is particularly important for experimental reproducibility and may enhance the reliability of this model for pharmacokinetic and toxicological evaluations.

Several limitations of the present study should be acknowledged. First, oxygen concentrations within the culture system were not directly measured. Although the improved ATP maintenance observed under shaking conditions is consistent with enhanced oxygen availability, this interpretation remains speculative without direct assessment of oxygen status or hypoxia-related markers. In addition, shaking culture may contribute to intratissue ATP preservation through multiple mechanisms, including improved nutrient delivery, waste removal, and mechanosensitive responses induced by medium circulation. Therefore, the precise mechanism underlying ATP preservation in kidney tissue slices remains unclear. Further studies using oxygen sensors or microelectrodes, together with evaluation of hypoxia-related markers such as HIF-1α, will be necessary to clarify the contribution of oxygen availability and other physical factors to energy homeostasis in kidney tissue slices. One additional limitation of the present study is that cultured slice viability was evaluated primarily based on intratissue ATP levels, whereas complementary cytotoxicity markers such as lactate dehydrogenase (LDH) release were not assessed. Although ATP content is widely used as an indicator of metabolic activity and tissue viability, additional evaluation of membrane integrity using LDH release assays would further strengthen the assessment of tissue injury and viability in cultured kidney tissue slices. Future studies incorporating multiple viability and cytotoxicity markers will therefore be necessary to further validate the culture system. Second, this study was performed using rat kidney tissue slices, and further studies are necessary to determine whether similar improvements can be achieved in human kidney tissue models. Despite these limitations, our findings demonstrate that shaking culture improves the maintenance of viability and functional activity in kidney tissue slices. This improved culture system may provide a useful experimental platform for studying renal drug transport, renal metabolism, and potentially DIKI *in vitro*. The metabolomic pathway results were visualized using the default raw p-value output from MetaboAnalyst, and FDR-adjusted values were also examined during interpretation. Given the inherent biological and technical variability in metabolomic datasets, these pathway findings should be considered exploratory and hypothesis-generating rather than definitive evidence of pathway dysregulation.

## Data Availability

The original contributions presented in the study are included in the article/Supplementary Material, further inquiries can be directed to the corresponding author.
