# Supplementary material for: Shaking culture improves physiological maintenance of primary rat kidney tissue slices
Source: Front Toxicol. 2026 Jun 25;8:1838970. doi: 10.3389/ftox.2026.1838970 (PMC13347121; doi:10.3389/ftox.2026.1838970)
Supplement: Supplementary file 1 [file DataSheet1.pdf]

Supplementary Material

Shaking culture improves physiological maintenance of primary rat kidney tissue slices

Moeno Kadoguchi[1], Kohei Matsushita[2], Jun Takahashi[3], Katsuhiro Esashika[3], Jingjing Yang[4], Masahiro Sugimoto[5], Ikumi Tamai[1], Hiroshi Arakawa[1,6*]

[1]Faculty of Pharmaceutical Sciences, Institute of Medical, Pharmaceutical and Health Sciences, Kanazawa University, Kakuma-machi, Kanazawa, Ishikawa 920-1192, Japan

[2]Division of Pathology, National Institute of Health Sciences, 3-25-26 Tono-machi, Kawasaki-ku, Kawasaki, Kanagawa 210-9501, Japan

[3]Advanced Materials & Solutions Research Laboratory, Research Center, Mitsui Chemicals, Inc., 1144 Togo, Mobara-shi, Chiba 297-0017, Japan

[4]Cell Culture Solution Department, New Business Incubation Center, Mitsui Chemicals, Inc., Tokyo Midtown Yaesu, Yaesu Central Tower 2-2-1, Yaesu, Chuo-ku, Tokyo 104-0028, Japan

[5]Institute for Advanced Biosciences, Keio University, Mizukami, Kakuganji, Tsuruoka, Yamagata 997-0052, Japan.

[6]Department of Regulatory Science, Graduate School of Pharmaceutical Sciences, Nagoya City University, 3-1 Tanabe-dori, Mizuho-ku, Nagoya 467-8603, Japan.

*** Correspondence:**
Hiroshi Arakawa, PhD
arakawa@phar.nagoya-cu.ac.jp

Supplementary Figures

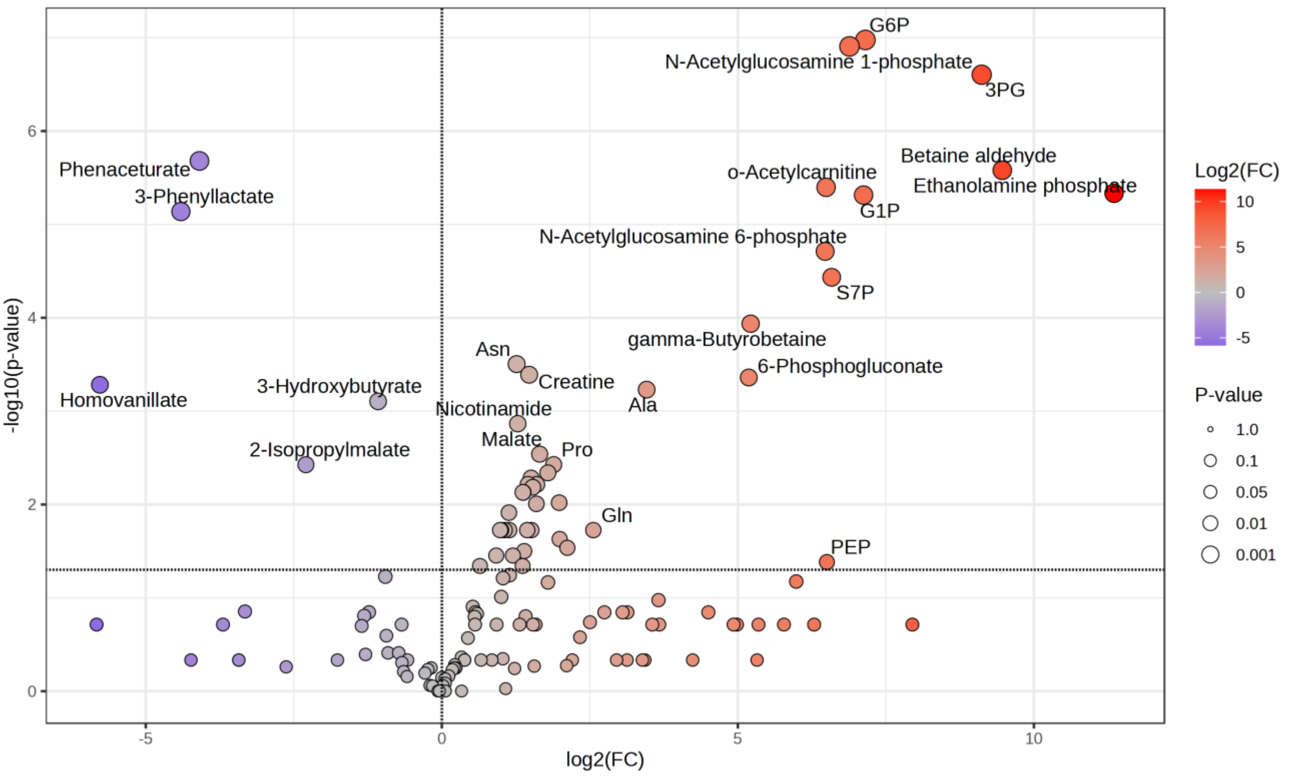

Supplementary Figure 1. Volcano plot analysis of metabolites comparing shaking culture and static culture conditions. Data were analyzed without sample normalization, with log10 transformation and auto scaling. Differential metabolites were evaluated using Student's t-test assuming equal variance with false discovery rate (FDR) correction for multiple comparisons. The x-axis represents log2 fold change (shaking culture/static culture), and the y-axis represents −log10(FDR-adjusted p-value).
